# Commentary: Vagal P2RY1 Receptors: A Novel Target for Airway Disease

**DOI:** 10.3389/fphar.2020.596003

**Published:** 2020-11-30

**Authors:** Juan Liu, Yu-Shi Hu, Yong Tang

**Affiliations:** ^1^ School of Sports Medicine and Health, Chengdu Sport University, Chengdu, China; ^2^ Sports Medicine Key Laboratory of Sichuan Province, Chengdu, China

**Keywords:** P2RY1 receptors, purinergic signalling, vagal sensory neurons, airway, protection

## Introduction

Recently, a paper published in *Cell* (Prescott et al., 2020) reported a novel role of purinergic P2RY1 receptors in vagal sensory neurons and their involvement in initiating a series of airway defense reflexes that guard the airways from external stimulus. This paper demonstrated how one of the rarely distributed laryngeal sensory neurons, P2RY1 neurons (∼100 neurons per mouse), can protect our airways to avoid pulmonary aspiration from ingested food and drinks.

The upper airway structures, including pharynx, epiglottis, larynx, and vocal folds, play an important role in regulating swallowing function, no matter if eating food or drinking liquids. These structures were intensively innervated by the vagus nerve through superior laryngeal (SLN) and recurrent laryngeal (RLN) nerve branches (Câmara and Griessenauer, 2015; Mazzone and Undem, 2016). Under physiological conditions, the vagus nerve usually initiates various defensive reflexes in the upper respiratory tract, e.g., transient apnea, pharyngeal swallow, vocal fold adduction, and expiratory reflexes, to protect the upper airway from some life-threatening invasions and avoid pulmonary aspiration. Failure of such vagally mediated defensive reflexes can be deadly in the clinic and can lead to some severe consequences, such as dysphagia (difficulty in swallowing), choking, speech impairment, weight loss, and aspiration pneumonia. The larynx is located within the anterior aspect of the neck, anterior to the inferior portion of the pharynx and superior to the trachea, with a total of six cartilages (three large unpaired cartilages: cricoid, thyroid, and epiglottis; three pairs of smaller cartilages: arytenoids, corniculate, and cuneiform) that are connected to each other by muscles and ligaments. Its main function is to protect the trachea by closing unexpectedly upon chemical or mechanical stimulations, thereby causing respiration suspension and preventing the penetration of foreign matter into the lower respiratory tracts (Waldman, 2009). Larynx also has other functions including vocal sounds production (phonation), coughing, the Valsalva maneuver, control of ventilation, and most importantly acting as a sensory organ. When external assault reaches to the larynx, a large range of defensive reflexive responses will be evoked by vagal laryngeal sensory neurons, eventually expelling these infiltrations out of the airways by a sudden cough (Steele and Miller, 2010; Mazzone et al., 2020). Previous studies have demonstrated that electric stimulations of vagus nerve could induce the characteristic airway protective reflexes including transient breathing pause, coughing, vocal fold adduction, and pharyngeal swallowing (Bolser, 1991). However, the sensory neuron pathways and molecular mechanisms are yet to be answered for better understanding of these sophisticated protection responses.

### Optogenetics Evoked Protective Reflexes in Laryngeal Sensory Neurons

In this paper, Prescott and Umans et al. firstly showed that pharyngeal swallow or expiratory reflexes can be evoked in mice under long-term anesthesia (urethane, 2 mg/g, *i.p.*). The evoked swallows or expiratory reflexes were determined mainly by visually observing the elevation of hyoid bone or a sudden forced airway withdrawal when giving different kinds of chemical or mechanical stimulations and did not occur during the saline perfusion. They found that the stimuli including water, acid, and high salt or mechanical stimuli by physical probing induced robust and repetitive pharyngeal swallows through the larynx (Yarmolinsky et al., 2009), while other laryngeal stimuli, like citric acid, saccharin, sucrose, monosodium glutamate, alanine, denatonium, quinine, allyl isothiocyanate, or capsaicin, all failed to evoke such pharyngeal swallows. Transection of the SLN abolished the swallowing responses to laryngeal water, acid, and high salt stimulation and reduced the mechanical force stimuli-induced swallows. Furthermore, they mimicked the fictive swallow after direct optogenetic activation of vagal sensory neurons by using a Cre-dependent channelrhodopsin allele (*loxP-ChR2*) to *Vglut2-ires-Cre* mice, which can specifically label the vast majority of sensory neurons in vagal and glossopharyngeal nerves. Optogenetic activation of vagal sensory neurons in nodose/jugular/petrosal (NJP) superganglia of *Vglut2-ires-Cre*; *loxP-ChR2* mice evoked an average of 4.3 pharyngeal swallows and occasional airway expiratory reflexes (1.5 events) per 10 s photostimulation trial.

### Mapping Vagal Sensory Neurons Subtypes

Optogenetic stimulation evoked swallow responses implying the involvement of laryngeal sensory afferents. Next, the authors applied single-cell RNA sequencing to delineate the vagal sensory neurons subtypes in the NJP ganglia (Mazzone et al., 2020). A total of ∼37 different classes of vagal/glossopharyngeal sensory neurons were identified by unsupervised clustering analysis, with 27 cell clusters (79%) derived from nodose and inferior petrosal ganglia and 10 cell clusters (21%) from the jugular and superior glossopharyngeal ganglia. Single-cell sequencing analysis revealed a vast majority of different signature genes were distributed in the 37 classes of NJP sensory neurons. Compared with *in situ hybridization* (ISH) validation of selected markers and the previous studies on identification of some vagal sensory neurons in controlling breathing, heart rate, blood pressure, and gut mobility (Chang et al., 2015; Williams et al., 2016; Min et al., 2019) they constructed remarkable sets of Cre-recombinase reporter mouse lines to target specifically genetic control of most of the 37 classified NJP sensory neuron subtypes. Together with self-developed or published mouse lines, 10 Cre-lines (*Vglut2-ires-Cre*, *Gabra1-ires-Cre*, *Gpr65-ires-Cre*, *Npy1r-Cre*, *Npy2r-ires-Cre*, *Calb1-ires-Cre*, *Piezo2-ires-Cre*, *Crhr2-ires-Cre*, *Glp1r-ires-Cre*, and *P2ry1-ires-Cre*) all crossed with *loxP-ChR2* were used to achieve targeting different small groups of sensory neurons in most of the 37 NJP neuronal populations (Chang et al., 2015; Williams et al., 2016; Nonomura et al., 2017; Zeng et al., 2018; Min et al., 2019). By using these amazing genetic Cre-lines toolkits, light-induced pharyngeal swallows were screened among the above mouse lines. Only by optogenetic stimulating vagal P2RY1 neurons evoked robust pharyngeal swallowing and expiratory reflexes, with 5.7 swallows and 3.0 events per 10 s light stimulation, though 11.6% of P2ry1 is expressed in NJP sensory neurons (∼250 neurons per ganglion) according to the prior ISH analysis. The pharyngeal swallows and expiratory reflexes were unable to be induced by light among other NJP sensory neurons, including Piezo2, Calb1, Gabara1, Crhr2, Gpr65, Npy2r, or Glp1r neurons. However, a small number of swallows and expiratory reflexes occurred by stimulating vagal Npy1r neurons. Airway defense response embodies multiple coordinated motor responses, not only pharyngeal swallowing and expiratory reflexes, but also transient apnea and vocal fold adduction to protect the airways. Using a miniaturized fiber endoscope or by the light endoscopy, optogenetic activation of vagal P2RY1 neurons evoked a full and complete glottic closure, which is deemed as the classical feature of airway protection program. Meanwhile, they also noticed that there was a short breathing pause accompanied with each swallow. However, the previous work by the author’s group also demonstrated that Piezo2, a mechanically activated cation channel, was involved in mediating airway stretch sensations and could evoke transient apnea as well (Zeng et al., 2018). They then employed two different genetically labeled mouse lines *P2ry1-GCaMP* (*P2ry1-ires-Cre; loxP-tdTomato; ROSA26-GCaMP3*) mice and *Piezo2-GCaMP* (*Piezo2-ires-Cre; loxP-tdTomato; ROSA26-GCaMP3*) mice, which express GCaMP3 from a constitutive promoter and Cre-expressing cells also express tdTomato in all vagal sensory neurons. Then they carried out single-cell calcium image to record vagal P2RY1 or Piezo2 neurons in response to stimulation of respiratory tract. Airway stretch induced calcium transients in most vagal sensory neurons of *Piezo2-GCaMP* mice (89.5%), while only 8.7% of sensory neurons of *P2ry1-GCaMP* mice showed calcium responses. These results suggest that vagal P2RY1 neurons mediate a full package of airway defense program, including pharyngeal swallowing, expiratory reflexes, vocal fold adduction, and transient apnea, against the harmful laryngeal challenges.

### Manipulating Vagal P2RY1 Sensory Neurons in Laryngeal Stimulations

Targeted ablation of vagal P2RY1 sensory neurons was done by direct injecting of diphtheria toxin (DT) into the vagal ganglia of *P2ry1-ires-Cre; loxP-DTR* mice (Buch et al., 2005). *In vivo* laryngeal perfusion of water, citric acid, high slat, and mechanical force was applied to vagal *P2ry1* ablation mice. Impaired swallowing responses to both water and citric acid stimulations were observed, with no changes to laryngeal force and high salt challenges. These findings provide evidence that laryngeal P2RY1 neurons are required for sensing specific chemosensory challenges in upper respiratory tract. Interestingly, for the unaffected high salt or force evoked swallows after P2RY1 ablation, it may indicate an alternative sensory pathway existed that helps our body to distinguish different kinds of airway assaults. Next, genetic-guided mapping results proved that vagal P2RY1 neurons indeed innervate the larynx and the terminals of P2RY1 neurons form a corpuscle-shaped structure around laryngeal taste buds. In order to do the mapping of vagal P2RY1 sensory neurons anatomical projections, the Cre-dependent Adeno-associated viruses (AAVs) encoding fluorescent (AAV-flex-tdTomato) or alkaline phosphatase (AAV-flex-AP) reporters were directly injected into NJP superganglia of *P2ry1-ires-Cre* mice, and labeled fibers were visualized by open-book wholemount images of these mice four weeks after injection. Previous study (Chang et al., 2015) demonstrated that vagal P2RY1 neurons are distributed in ciliated epithelium, including the epiglottis and subglottis, arytenoid cartilages, and aryepiglottal folds. Taste cell marker KRT8 (Boggs et al., 2016) immunohistochemical staining showed that P2RY1 neurons terminals also directly reach to laryngeal taste buds in squamous epithelium in a ∼40 *μ*m diameter corpuscle-like structure.

**FIGURE 1:**
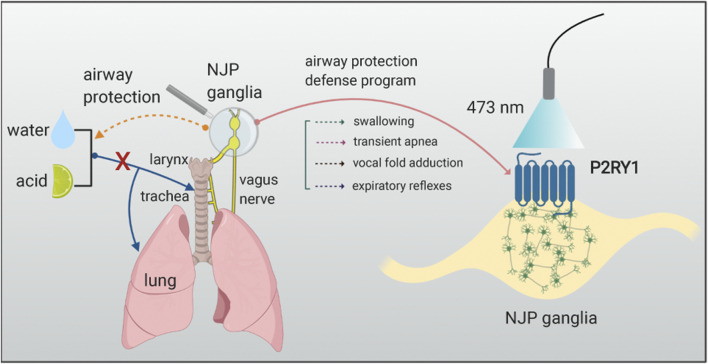
Vagal sensory P2RY1 neurons mediated airway protection reflexes. The graph illustrates that activation of P2RY1 neurons in the NJP superganglia evokes a full set of laryngeal protective reflexes responses. P2RY1 are expressed in a small population of vagal sensory neurons (∼100 neurons/mouse). Blue light activation of these vagal sensory P2RY1 neurons can induce the following protective responses such as pharyngeal swallowing, expulsion reflex, transient apnea, vocal fold adduction, and hyoid bone elevation. These reflexes are helpful to defend choking, dysphagia, cough, or other respiratory symptoms when the water or acid stimulations enter the airway system.

Since some P2RY1 neurons terminals also contacted taste buds in the oropharynx and lingual taste buds in foliate and circumvallate papillae (Yarmolinsky et al., 2009), P2RY1 neurons function in these structures also needed to be elucidated. It is interesting to explore how upstream sentinel cells in the epithelium respond to laryngeal acid and water challenge. The research team again applied genetic method to generate *Krt8-Cre* ^*ER*^ *; loxP-ChR2* mice, which respectively, target laryngeal taste buds within squamous epithelium, but not vagal sensory neurons. Employing optogenetic stimulation directly toward the inferior edge of the arytenoids and vocal folds, robust swallows were observed after light illumination, but not stimulating the NJP ganglia in *Krt8-Cre* ^*ER*^ *; loxP-ChR2* mice. These findings again support that P2RY1 neurons function as downstream of the epithelial cells in the larynx and receive information and react to the corresponding chemical or mechanical stimulations. In the end, the ATP signaling was explored in this study based on the previous finding that ATP works as neurotransmitter mediating communications between lingual taste cells and second-order gustatory fibers (Finger et al., 2005; Takahashi et al., 2016). Knockout of both the P2X2 and the P2X3 receptors mice showed a complete loss of laryngeal water sensation, a reduction in acid stimulation, and intact response to mechanical force and high salt stimulations. Together, these data demonstrate that epithelial sentinel cells firstly detect some certain chemical stimulations and then pass the information to vagal sensory P2RY1 neurons via ATP, eventually inducing a range of defensive responses program, including transient apnea, vocal fold adduction, pharyngeal swallow, and expiratory reflexes (Figure 1).

## Discussion

This study (Prescott et al., 2020) provides a promising role of purinergic P2RY1 receptors in vagal sensory neurons that function as a second-order sensory neuron to detect airway threats and coordinate defensive reflexes to guard airway integrity. The experimental methodology including a large deal of genetically labeled Cre-mouse lines and single-cell RNA sequencing for broadly investigating the sensory molecular diversity and specific targeting strategy or optogenetic regulation for specific sensory neurons and cell type specific ablation based on Cre-dependent DT receptor (DTR) all help to illustrate the cellular diversity and mechanisms of vagal and glossopharyngeal nerves.

Many questions and future research directions arise from the current discovery of vagal P2RY1 sensory neurons in mediating respiratory defense reflexes. First, both water and citric acid induced pharyngeal swallows after optogenetic stimulating of P2RY1 sensory neurons (Moayedi et al., 2020), while further study of the transducer cells of water-sensing and acid-sensing neurons in the larynx need to be elucidated. Whether other cell types such as laryngeal taste cells, solitary chemosensory cells, neuroendocrine cells, or epithelial or immune cell types may play an important role in mediating these tastes’ sensation needs to be addressed (Yarmolinsky et al., 2009). Second, how the different sensation detected by P2RY1 sensory neurons in the larynx relays information to upper central nervous system in the brain and the output pathway in initiating rapid motor responses needs to be addressed (Figure 1). The nucleus tractus solitarius, also known as nucleus of the solitary tract in the brainstem, which impacts on many homeostatic systems within the body (King, 2007), has been recognized as an essential relay station for primary visceral sensor within the brain which communicates between the viscera and brain axis (King, 2007; Scherrer et al., 2009; Stanley et al., 2010; Zoccal et al., 2014). It receives and responds to stimuli from the respiratory, cardiovascular, and gastrointestinal systems. The recombinant virus-tracing technology by using the retrogradely transported neurotropic pseudorabies virus or anterogradely transported herpes simplex virus 1, strain H129 (HSV-1-H129) could help to dissect viscera-brain connectivity pathways or the viscera-brain neuronal circuits (Schwarz et al., 2015; Fan et al., 2020). Third, single-cell RNA sequencing data revealed a great number of different molecular verified neuron subtypes in the NJP superganglia; instead, most of their functions in vagal sensory neuron are not fully studied. Finally, it would be also interesting to study the electrophysiological properties of P2RY1 sensory neuron in the NJP ganglia, which would help to understand P2RY1 neurons in mediating airway protection response under physiological or pathological conditions in a more comprehensive way.

Another issue that needs to be considered is the influence of P2RY1 development difference on vagal P2Y1 sensory protection function. Studies showed that there’s age difference of P2R1 mRNA expression in microglia cell culture from 3 days to 4 months (Crain et al., 2009). P2Y1 expression of 21-day-, 7-week-, and 4-month-old C57 male mice showed increased expression (by 50–100-fold) when compared to 3-day-old male mice. Female mice also showed similar results of P2RY1 mRNA expression pattern as male mice. No sexual dimorphisms of P2RY1 receptors expression in microglia were observed. However, it would be interesting to explore the P2RY1 receptors expression in vagal sensory neurons as well as the sex difference. Human study revealed that air or water stimulation can induce healthy neonates to have a 30 or 76% pharyngeal reflexive swallowing response, respectively (Jadcherla et al., 2007). However, the development differences of age or sex of vagal P2RY1 sensory neurons are unknown and how they affect the swallowing ability is still unclear. It would be meaningful to check the vagal P2RY1 sensory neurons function between young and old mice. This can be helpful to explain why both newborn and old patient are more at risk of suffering from dysphagia in the clinic. Furthermore, this vagal sensory neuron function mediated by P2Y1R might be involved in the role of vagal activity and airway function in diseases such as asthma and chronic obstructive pulmonary disease (COPD). Chang et al. showed that optogenetic activation of P2RY1 neurons acutely silence respiration, trapping animals in exhalation (Chang et al., 2015). P2RY1 neurons did not impact heart rate or gastric pressure, other autonomic functions. Shortness of breath is a common symptom of asthma and COPD patients in the clinic. It would be intriguing to explore how P2RY1 contributes to alleviating the symptom of asthma and COPD. Study from Tränkner et al. showed that ablation or silence of vagal TRPV1 expressing sensory neurons abolished the hyperreactive bronchoconstriction after a fully developed lung inflammatory immune response by ovalbumin stimulation (Tränkner et al., 2014). Optogenetic stimulation of TRP expressing cells dramatically exacerbated airway activity of inflamed airways. The sphingosine-1-phosphate receptor 3 was coexpressed with TRPV1 neurons. Calcium images demonstrated that capsaicin activated 60.7% of all vagal sensory neurons, but none of P2RY1 neurons by nodose/jugular ganglia acute cultures (Chang et al., 2015). ISH analysis showed that 95% of P2RY1 neurons did not express TRPV1 in vagal sensory neuron. These results suggest that the immune function may be through glia but not sensory neuron P2RY1 receptor, which still need to be elucidated.

Overall, a small cluster of P2RY1 receptors in vagal sensory neuron mediates a series of stereotyped airway protection programs. Moreover, P2RY1 neurons appose laryngeal taste buds in a corpuscular-like structure, which receive signal elicited from ATP signaling in epithelial sentinel cells. More exciting discoveries of vagal P2RY1 sensory neurons will be explored with further understanding of P2RY1 receptor and structure function studies (Zhang et al., 2015). Advanced techniques and methods, such as optogenetics, chemogenetics, *in vivo* calcium imaging, fiber photometry, virus tracing, will facilitate our understanding of the diversity of vagal P2RY1 sensory neurons in guarding the respiratory tract from external assaults and its central mechanism. Hence, increasing P2RY1 functional studies will help to shed lights on promoting the development of pharmaceutical drugs against swallowing disorders related diseases such as dysphagia and aspiration pneumonia in the clinic.

## Author Contributions

JL, YH, and YT drafted the manuscript. All authors contributed to manuscript revision and read and approved the submitted version.

## Funding

Our work was supported by the National Natural Science Foundation of China (81904312).

## Conflict of Interest

The authors declare that the research was conducted in the absence of any commercial or financial relationships that could be construed as a potential conflict of interest.
